# The Effect of Antihelminthic Treatment on Subjects with Asthma from an Endemic Area of Schistosomiasis: A Randomized, Double-Blinded, and Placebo-Controlled Trial

**DOI:** 10.1155/2012/296856

**Published:** 2012-08-13

**Authors:** Maria Cecilia F. Almeida, Givaneide S. Lima, Luciana S. Cardoso, Robson P. de Souza, Régis A. Campos, Alvaro A. Cruz, Joanemile P. Figueiredo, Ricardo R. Oliveira, Edgar M. Carvalho, Maria Ilma Araujo

**Affiliations:** ^1^Serviço de Imunologia, Universidade Federal da Bahia (UFBA), 40110-160 Salvador, BA, Brazil; ^2^Departamento de Ciências da Vida, Universidade do Estado da Bahia UNEB, 41.150-000 Salvador, BA, Brazil; ^3^Instituto Nacional de Ciências e Tecnologia em Doenças Tropicais (INCT-DT/CNPq-MCT), Brazil; ^4^ProAR, Núcleo de Excelência em Asma, Universidade Federal da Bahia 40110-160 Salvador, BA, Brazil; ^5^Faculdade de Farmácia, UFBA 40110-160 Salvador, BA, Brazil; ^6^Escola Bahiana de Medicina e Saúde Pública, EBMSP, Salvador, Bahia, Brazil

## Abstract

This is a prospective, double-blinded, and placebo-controlled trial evaluating the influence of antihelminthic treatments on asthma severity in individuals living in an endemic area of schistosomiasis. Patients from group 1 received placebo of Albendazole or of Praziquantel and from group 2 received Albendazole and Praziquantel. Asthma severity was assessed by clinical scores and by pulmonary function test. There was no significant difference in the asthma scores from D0 to D1–D7 after Albendazole or Praziquantel and from D0 to D30–90 after Albendazole or Praziquantel in both, group 1 and 2. It was observed, however, a clinical worsening of the overall studied population after 6 months and 12 months of antihelminthic treatments. Additionally, we observed increased frequency of forced expiratory volume in 1 second (FEV1) <80% on 12 and 18 months after treatment. The worsening of asthma severity after repeated antihelminthic treatments is consistent with the hypothesis of the protective role conferred by helminths in atopic diseases.

## 1. Introduction

Helminthic infections and allergic diseases are highly prevalent in many parts of the world, and both lead to type 2 immune response with secretion of IL-4, IL-5, and IL-13, with a consequent increase in the production of IgE and eosinophilia. Lynch et al. [1] studying the skin prick test (SPT) response in children from an ascariasis endemic area observed a decreased reactivity to the test and subsequently an increased response after treatment of intestinal helminths [2].

Studies conducted by the Immunology Service (SIM) of the Federal University of Bahia, Brazil, demonstrated a negative association between the cutaneous immediate hypersensitivity response to the skin prick test to aeroallergens and *Schistosoma mansoni *parasite load, measured by a quantitative assessment of the number of eggs per gram of stool [3]. Over a year-long follow-up study in the state of Bahia, a group of researchers from SIM compared three populations from impoverished areas of the state, including one group from an endemic area of *S. mansoni*, one group from a rural nonendemic area, and a third group from a slum area in Salvador, the capital of the state. In the study, it was observed that asthma severity indicators were lower in individuals living in the endemic areas of schistosomiasis when compared to the other two areas in which there were no recorded cases of *S. mansoni* transmission [4]. 

Several factors may explain the lower frequency of positive SPT and lower asthma severity in populations infected with helminths. The possible hypothesis includes high production of polyclonal IgE and reduced levels of allergen-specific IgE [1, 2], high concentrations of antigen-specific IgG4 [5], activation of regulatory cells, and regulatory cytokine production [6]. For instance, IL-10 can promote a decrease in the release of histamine and other mast cell mediators [7]. Since infection by *S. mansoni* induces high production of IL-10, it is possible that this is the main mechanism by which the allergic response is suppressed in infected individuals. This hypothesis is reinforced by studies of van den Biggelaar et al. [6], which showed that reduced reactivity to SPT in African children infected with *S. haematobium* was associated with an increase in IL-10 production *in vitro* by cells of these individuals. Our hypothesis for the current study is that treatment of helminth infections, including a drug to treat schistosomiasis, alters the immune response leading to worsening in asthma outcomes. The aims of this study were, firstly, evaluating early events associated to asthma severity resulting from treatment of helminth infections, and secondly, determining the degree of interference of antihelminthic treatment in the clinical course of asthma in a randomized, double-blinded, and placebo-controlled trial. 

## 2. Materials and Methods

### 2.1. Subjects and Study Design

This is a randomized, double-blinded, and placebo-controlled trial with two groups of asthmatics living in a *S. mansoni* endemic area. The study was carried out in Água Preta, a small village near the city of Gandu in the State of Bahia in Brazil. Gandu is located 280 km south of Salvador, the capital of the State of Bahia. Água Preta is composed of a residential area in the center of the village and surrounding farms. There are approximately 800 people living in that community. They live in poor sanitary conditions and agriculture is their predominant occupation. The Água Preta community was identified by our group as a community with a frequent infection of *Schistosoma mansoni* (49.5% in a survey of 427 residents in 2006) and helminthic infection in general (prevalence of *Ascaris lumbricoides*, *Trichuris trichiura*, and hookworm at that time was 24.2%, 33.8%, and 22.2%, resp.). Six hundred one individuals from Água Preta agreed to participate in this study. They were screened for asthma through a direct questionnaire on the basis of the International Study of Asthma and Allergies in Childhood (ISAAC) [8]. In addition, questions concerning concomitant illnesses, socioeconomic status, and living conditions were asked in a complementary questionnaire. Patients were selected as having asthma if their responses to the ISAAC questionnaire were considered by a physician to be indicative of a personal history of asthma in the past 12 months, and if they were 5 to 50 years old. Children under 5 years old were not included due to difficulties in performing the pulmonary function test. Subjects over 50 years old were not included due to increased rates of chronic obstructive pulmonary disease in this age group. Only fifty asthmatic individuals from the community met the inclusion criteria and they were randomized either as group 1 (who would receive initially placebo of antihelminthic treatments; *n* = 25) or group 2 (who would receive Albendazole in a split dose of 400 mg, followed by Praziquantel 50 mg/Kg of body weight a week later; *n* = 25). Five individuals from group 1 left the study in the first week after the beginning of the study for personal reasons, remaining 20 individuals in this group. The loss of these individuals did not alter significantly demographic features of group 1, such as the age and gender distribution. At each evaluation, every patient underwent a physical examination, always performed by the same physician (blind to the type of treatment). At the evaluations, common asthma symptoms and signs were checked, such as cough, dyspnea, and wheezing. In addition, a questionnaire elicited information on asthma (i.e., presence and frequency of asthma attacks, type of treatment received during the attack at home, emergency department, or hospital) and the use of prophylactic or symptomatic antiasthma drugs (e.g., antihistamine, inhaled, or oral beta-2-agonist, and inhaled, oral, or parenteral corticosteroid) since the previous evaluation. These parameters were scored as follows: physical examination as 0 (normal examination) and 1 (at least one abnormal finding); asthma exacerbations as 0 (no), 1 (yes, if treated at home), and 2 (yes, if treated at an emergency department or at a hospital); use of antiasthma drugs as 0 (no), 1 (yes, except for oral or parenteral corticosteroid use), and 2 (yes, if oral or parenteral corticosteroid had been used) as reported in a previous study [4]. 

Human subject study guidelines of the US Department of Health and Human Service were followed in the conduction of this study. The study was approved by the Ethics Committee of Professor Edgard Santos Hospital. Informed consent was obtained from all patients or their legal guardians.

**Figure 1:**
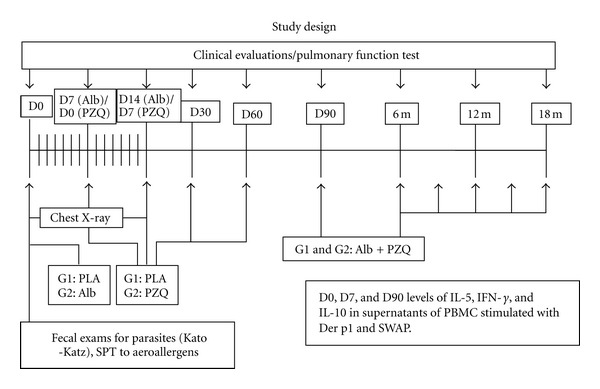
Flowchart of the study design.

In the initial assessment, patients responded to a questionnaire to assess the clinical score of asthma [4] underwent a physical examination, chest radiograph, pulmonary function test, and blood sampling for evaluation of immune response (IL-5, IL-10, and IFN-*γ* in supernatants of cultures stimulated with SWAP and Der p1). Stool exams for parasites were also performed on each patient. The two groups were then treated with antihelminthics or placebo and reevaluated after the completion of treatment as shown in the study design flowchart (Figure 1). Chest radiographs and pulmonary function tests were performed at the time of enrolment and at day 7 after treatment. Spirometry test was repeated 30 days after treatment. Immunological evaluation was performed at enrolment and repeated at 7 and 90 days after treatment. After 90 days of enrolment, both placebo and treatment groups were treated with Albendazole and Praziquantel. From there, the two groups were assessed monthly by clinical examination, questionnaire, and pulmonary function test for a total period of 18 months according to the study design (Figure 1).

### 2.2. Pulmonary Function Tests, Chest X-Ray, and Skin Prick Test to Aeroallergens (SPT)

Pulmonary function tests were performed on all subjects at enrollment and at each visit thereafter. The parameter used was Forced Expiratory Volume in 1 second (FEV1). Results were considered as normal when FEV1 value was ≥80% [9]. 

Chest X-ray was performed in a specialized clinic in Gandu, at baseline and at D7 after Praziquantel treatment. SPTs were performed on the right forearms of all individuals at enrolment using *Dermatophagoides pteronyssinus* (Der p), *D. farinae* (Der f), *Blomia tropicalis* (Blo t), *Periplaneta americana* (Per a), and *Blattella germanica* (Bla g) glycerinated allergen extracts (FDA Allergenic). Histamine (1 : 1000) and glycerinated saline were used as positive and negative controls, respectively. A positive skin reaction was defined as formation of a wheal with a mean diameter greater than 3 mm. The SPT results were read 20 minutes after application, and a SPT response was considered as positive if there was at least one positive test of the five tested allergens.

### 2.3. Immune Response—Cell Culture and Cytokine Measurements

All enrolled individuals had their blood taken for immunological studies. Peripheral blood mononuclear cells (PBMC) from the blood samples of the two groups were analyzed for *in vitro* immune response, which included measurement of IL-10, IL-5, and IFN-*γ* production by PBMCs in response to soluble *S. mansoni* adult worm antigen (SWAP, kindly provided by Dr. Alfredo Góes from the Federal University of Minas Gerais, Brazil) and to the antigen 1 from *D. pteronyssinus* (Der p1 extract, cosmo Bio Co., Ltd.). 

PBMCs from individuals of the study were obtained through the Ficoll-Hypaque gradient and adjusted to the concentration of 3 × 10^6^ cells/mL in complete RPMI medium (Life technologies GIBCO BRL, Gaithersburg, MD). Cells were cultured *in vitro* with the antigens Der p1 (25 *μ*g/mL) and SWAP (10 *μ*g/mL). The mitogen phytohemagglutinin (PHA) in the final concentration of 2 *μ*g/mL was also used in the cultures. The cultures were incubated for 72 hours at 37°C and 5% CO_2_ and the supernatants were collected for cytokine measurements. Levels of IL-5, IL-10, and IFN-*γ* were determined by ELISA sandwich technique, using commercially available kits (R&D Systems) and the results were expressed in picograms per milliliter based on a standard curve.

### 2.4. Fecal Examinations for Parasites

Three stool samples from each individual were examined using the Hoffman sedimentation method to identify helminths and enteric protozoa, and the Kato-Katz method was used to estimate parasite load [10].

### 2.5. Sample Size and Statistical Analysis

Only fifty asthmatic individuals from the village where the study was carried out met the inclusion criteria. The power of the study was calculated taking into account the results of a previous study from our group [4] which demonstrated that the frequency of asthma symptoms in asthmatic individuals living in an endemic area of schistosomiasis is 18.6%, whereas the prevalence of symptoms in a worm-free population is 58.7%. Based on these data, a sample size of 20 in the placebo group and 25 in the treated group has 76% power to detect a difference between proportions with a significance alpha level of 0.05 (two-tailed).

Statistical analyses were performed using the software Statistical Package for Social Science (version 9.0 for Windows; SPSS). Fisher's exact test was used to compare proportions. The Mann-Whitney *U* test was used to compare levels of cytokines between groups, and the Wilcoxon matched-pairs signed rank test was used to compare the levels of cytokines intragroup before and after antihelminthic treatments. Statistical significance was established at the 95% confidence interval.

## 3. Results

### 3.1. Features of the Studied Subjects

The study included 45 asthmatic patients. They were divided into two groups: one group received placebo of antihelminthic treatments (placebo of Albendazole and placebo of Praziquantel (group 1 or placebo group), while the other group received Albendazole to treat geohelminths and Praziquantel to treat *S. mansoni*  infection (group 2 or Praziquantel group). The demographic data of subjects enrolled in the study are shown in Table 1. *S. mansoni* parasite burden and infection with other helminths as well as the frequency of positive skin prick test to aeroallergens are also shown in Table 1. 

Gender distribution did not differ significantly between groups who received placebos (G1; 50% male) and those who received antihelminthic treatment (G2; 32% male, *P* > 0.05; Table 1). 

The mean age of patients included in the study was 17.6 ± 13.1 years. There was no significant difference in the mean age between groups (21.3 ± 15.4 and 14.7 ± 10.3 years in Group 1 and Group 2, resp.) with age ranges from 6 to 20 years found in 65% of G1 and in 72% of G2 (*P* > 0.05) and 21 to 50 years in 35% and 28% of G1 and G2, respectively (*P* > 0.05; Table 1). 

The frequency of rhinitis did not differ significantly between patients from group 1 and 2, being 50% and 72%, respectively (*P* > 0.05; Table 1). The frequency of active smokers was similar between the placebo and Praziquantel groups (30% and 28%, resp.; *P* > 0.05), while the frequency of second-hand smoking exposure was higher in patients from group 1 (90%) then in group 2 (36%; *P* < 0.0005; Table 1).

There was no significant difference in the frequency of FEV1 ≤80% between the placebo and Praziquantel groups at baseline (5% and 12%, resp.; *P* > 0.05). A positive response to the skin prick test to aeroallergens was found in 36% of patients from the Praziquantel group compared to 45% of placebo group at baseline (*P* > 0.05; Table 1). The frequency of positive response to the different aeroallergens such as Der p, Der f, Blo t, Per a and Bla g also did not differ between the two groups of patients (*P* > 0.05). A positive response to histamine was found in 100% of patients from group 1 and 90.9% of those from group 2 (*P* > 0.05; Table 1).

The frequency of *S. mansoni* infection did not differ between groups (*P* > 0.05). The *S. mansoni* parasite burden also did not differ significantly between group 1 and group 2 (115 ± 49 and 427 ± 228 eggs/g feces, resp.; *P* > 0.05). Patients were also infected with other helminths, such as *Ascaris lumbricoides*, *Trichuris trichiura*, and hookworm. However, there was no significant difference in the frequency of other helminthic infection between groups (Table 1; *P* > 0.05). Co-infection with *S. mansoni* and one or more other helminths were observed in 90% and 100% of patients from G1 and G2, respectively (*P* > 0.05; Table 1).

**Figure 2:**
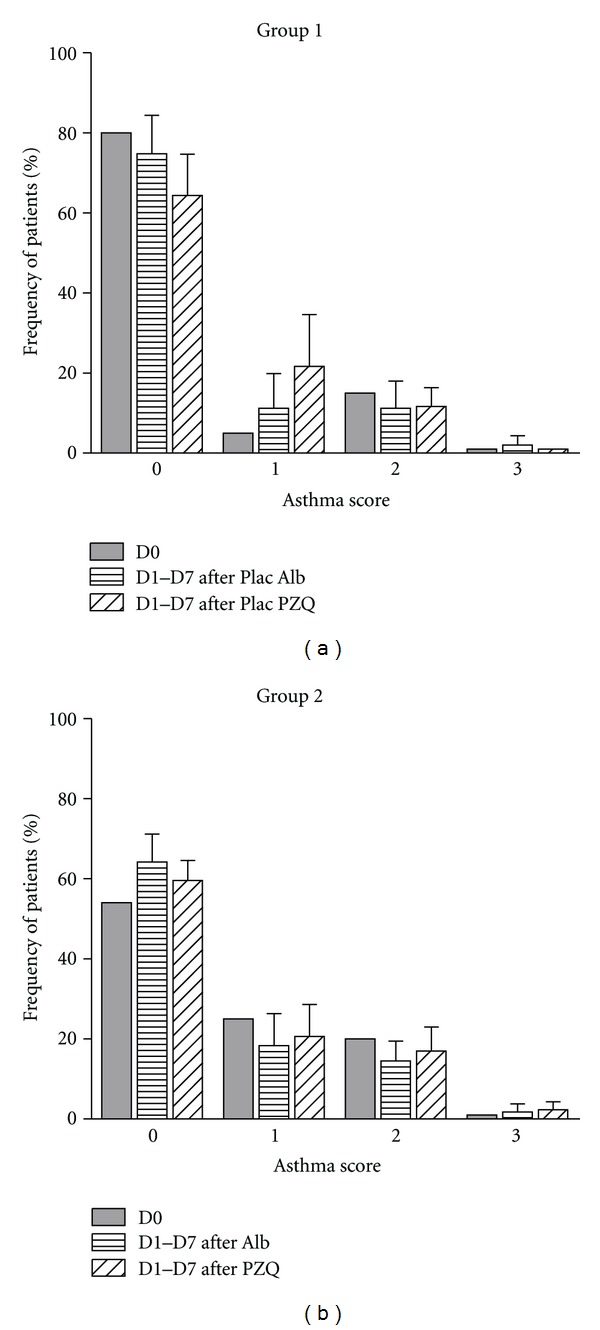
Frequency of clinical scores of asthma in patients from Group 1 at day zero (D0) and one to seven days (D1–D7) after treatment with placebo (Plac) of Albendazole (Alb) and placebo of Praziquantel (PZQ) (a); and at day zero (D0) and one to seven days (D1–D7) after treatment with Albendazole or Praziquantel (Group 2) (b). Praziquantel was given seven days after the treatment with Albendazole in the Group 2. Data were represented as mean ± SD. There was no significant difference in the frequency of asthma scores between D0 versus D1–D7 in none of groups (*P* > 0.05; Chi-square Test for Independence).

Clinical scores for asthma were initially evaluated at baseline (day 0 or pretreatment) and for seven consecutive days during the first week after treatment with placebo or Albendazole (D1 to D7). At day 7 after Albendazole treatment, patients from group 1 and group 2 were treated with placebo of Praziquantel and Praziquantel, respectively. They were clinically evaluated and had their asthma score recorded during the following seven days. The results of clinical scores of asthma after treatment with placebo of Albendazole and placebo of Praziquantel are shown in Figure 2(a). There was no significant difference in frequency of asthma scores at D1 to D7 compared to D0 (*P* > 0.05) in patients from group 1 who received placebos of Albendazole and Praziquantel (Figure 2(a)).

There was also no significant difference in the frequency of asthma scores from D0 to D1–D7 after Albendazol and Praziquantel in group 2 (*P* > 0.05; Figure 2(b)).

**Figure 3:**
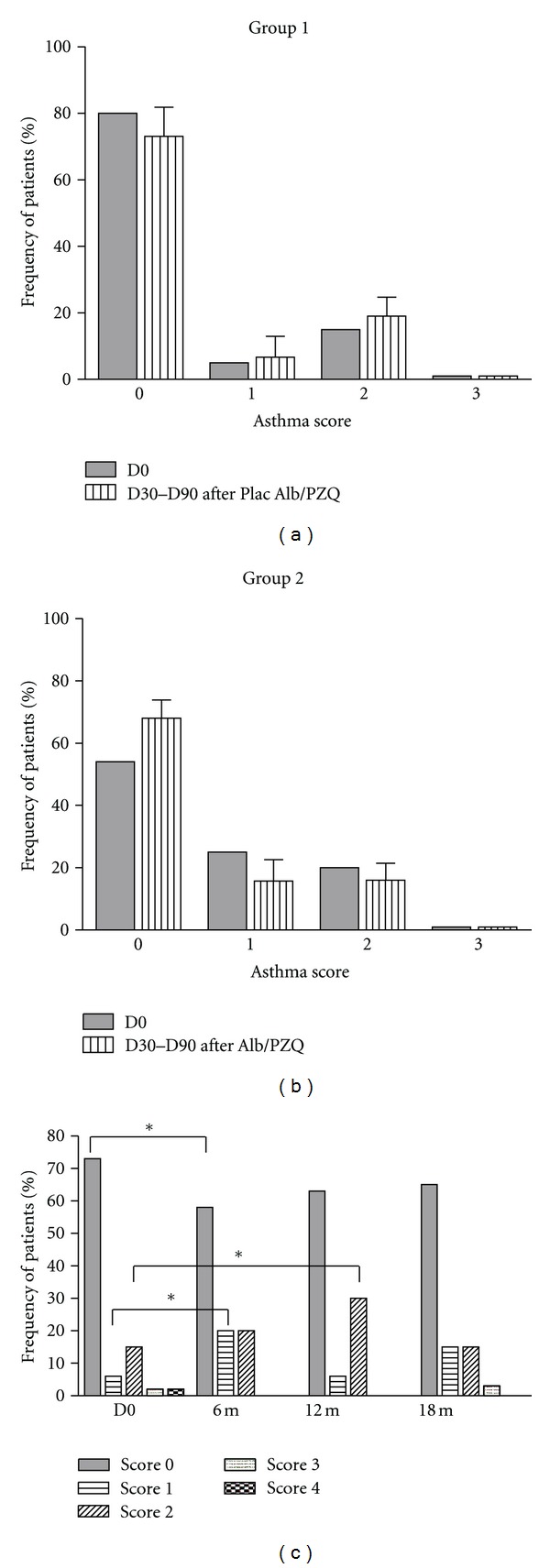
Frequency of clinical scores of asthma at day zero (D0) and day 30 to day 90 (D30–D90) after treatment with placebo (Plac) of Albendazole (Alb) and placebo of Praziquantel (PZQ) (Group 1; (a)); and frequency of clinical scores of asthma at day zero (D0) and day 30 to 90 (D30–D90) after treatment with Albendazole and Praziquantel (Group 2; (b)). Data were represented as mean ± SD. (c) shows the frequency of clinical scores of asthma (0, 1, 2, 3, 4) at day zero (D0) and at 6 months, 12 months and 18 months after treatment with Praziquantel (Figure 3(c)). Patients were evaluated monthly from day 30 to day 90 (D30–D90). After 90 days of enrollment, both placebo and treatment groups were treated with Albendazole and Praziquantel. From there clinical scores of asthma were assessed each 6 months for a total period of 18 months. *D0 versus 6 m or D0 versus 12 m, *P* < 0.05; Fisher's exact test.

The mid-term effect of Praziquantel treatment on the asthma severity was evaluated during the first 90 days of posttreatment. Three monthly consecutive evaluations (D30 to D90) were performed in each patient in this time period. The mean frequencies of asthma clinical scores are shown in Figure 3(a). There was no significant difference in the frequency of clinical scores 0, 1, 2, 3, or 4 between D0 (80%, 5%, 15%, 0%, 0%) and D30 to D90 (73 ± 8.8%, 6.7 ± 6.2%, 19 ± 5.7%, 0%, 0%) in the placebo group (Figure 3(a)). In the group treated with Praziquantel, the frequency of different scores (0, 1, 2, 3, and 4) also did not differ from D0 (55%, 25%, 20%, 0%, 0%) to D30–D90 (68 ± 5%, 15.7 ± 6.9%, 16 ± 5.4%, 0%, 0%; *P* > 0.05; Figure 3(b)).

The long-term effect of antihelminthic treatment on asthma severity was evaluated in three different time periods, 6, 12, and 18 months after-treatments. The placebo group was treated with Albendazole and Praziquantel at day 90, when the Praziquantel group received the second treatment with these two drugs. Since, after day 90, both groups were treated with antihelminthics, the groups were combined into one group of treated patients thereafter. The frequency of score zero in the studied population was lower after 6 months of treatment (58%) compared to D0 (73%; *P* < 0.05; Figure 3(c)), being the frequency of score one higher (20%) compared to baseline (6%; *P* < 0.05). Likewise, the frequency of score 2 was higher after 12 months of treatment (30%) compared to D0 (15%; *P* < 0.05, Figure 3(c)).

There was, however, no significant difference in the frequency of different asthma clinical scores at 18 months after treatments (65%, 15%, 15%, 3%, 0%, to scores 1, 2, 3, and 4, resp.) compared to baseline (73%, 6%, 15%, 2% and 2% to scores 1, 2, 3, and 4, resp.; *P* > 0.05; Figure 3(c)).

**Figure 4:**
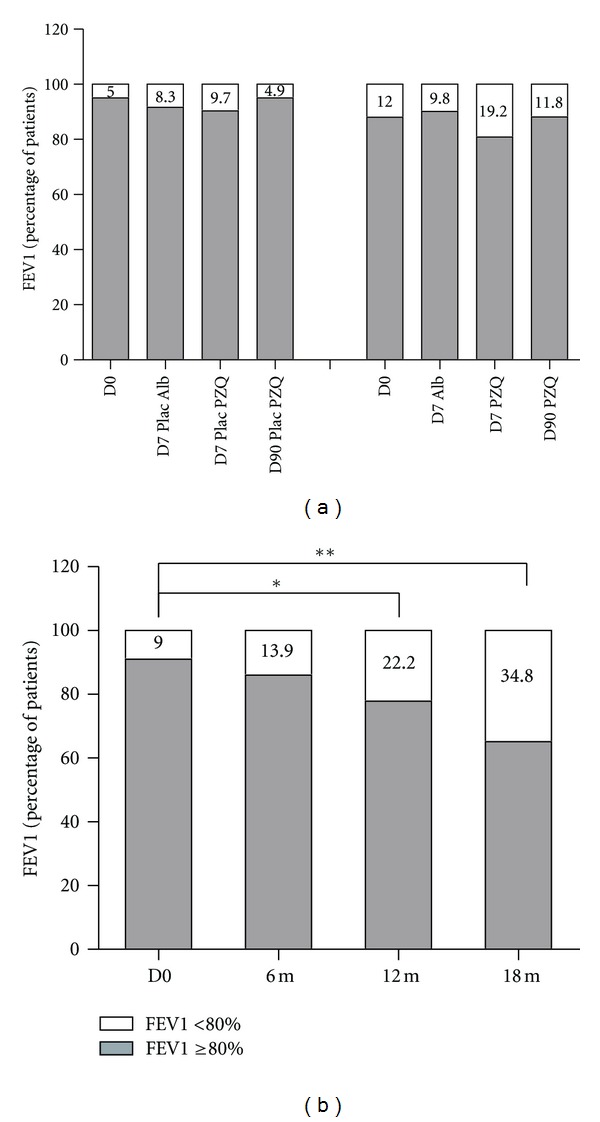
Frequency of Forced Expiratory Volume in 1 second (FEV1) <80% at D0, D7 after placebo of Albendazole treatment and D7 and D90 after placebo of Praziquantel treatments in the Group 1 and after treatments with Albendazole and Praziquantel in the Group 2 (Figure 4(a)). Frequency of FEV1 <80% at day zero (D0) and 6, 12 and 18 months after treatment with Albendazole and Praziquantel (b). Results were considered as normal when FEV1 value was ≥80%. Numbers in the top of the bars represent the percentage of patients who had FEV1<80%. *D0 versus 12 m, *P* < 0.05; **D0 versus 18 m, *P* = 0.0001; Fisher's exact test.

 The severity of asthma was also evaluated through pulmonary function test (PFT). The result of FEV1 in patients from group one and two are shown in Figure 4. There was no significant difference in the frequency of FEV1 <80% either in Praziquantel or in the placebo groups when the baseline values were compared to D7 post Albendazole, D7 after Praziquantel and also to D90 after Praziquantel (*P* > 0.05, Figure 4(a)). On the other hand, the frequency of FEV1 <80% among all the subjects with asthma (*n* = 45) was higher at 12 months (22.2%) and subsequently at 18 months after treatment (34.8%) compared to the frequency at baseline (9%; *P* < 0.05, Figure 4(b)). 

### 3.2. Cytokine Profile Induced by *S. mansoni* and *D. pteronyssinus* Antigens in PBMCs of the Studied Population

We measured the cytokines IFN-*γ*, IL-5, and IL-10 in the supernatants of PBMC cultures stimulated with the *S. mansoni* soluble adult worm antigen (SWAP) and the antigen 1 of the *D. pteronyssinus* (Der p1). There was no significant difference in the levels of IFN-*γ* between D0 and D7 or D0 and D90 in response to SWAP and Der p1 (*P* > 0.05, Table 2). The baseline of IL-5 mean levels in response to Der p1 in cultures was lower in the placebo group (150 pg/mL) than in Praziquantel group (463 pg/mL; *P* < 0.05). There was, however, no significant difference between the baseline mean values and those found at D7 or D90 after treatment in response to SWAP or Der p1 in either group (*P* > 0.05; Table 2). On the other hand, in the group of Praziquantel, the mean levels of IL-10 in response to SWAP decreased from 642 pg/mL at D0 to 175.6 pg/mL at D90 after treatment (*P* < 0.05; Table 2). There was no significant difference in the mean level of IL-10 in response to Der p1 comparing baseline values with those from D7 and D90 in both placebo and Praziquantel groups (Table 2). There was a high production of IL-10, IL-5, and IFN-*γ* in cultures stimulated with the mitogen PHA compared to nonstimulated cultures (*P* < 0.05) in both groups of patients (data not shown).

## 4. Discussion

The aim of this study was to determine in a randomized, double-blinded, and placebo-controlled trial whether antihelminthic treatment would interfere with the clinical course of asthma in individuals living in a *S. mansoni* endemic area. Additionally, we evaluated the *in vitro* cytokine profile in response to an aeroallergen and to *S. mansoni* antigen before and after antihelminthic treatment. There was no change in the asthma score during the first weeks after treatment in both groups, neither was there any variation after 30 to 90 days. At day 90, group 1 and group 2 were combined into one group who received Albendazole and Praziquantel each three months thereafter. When comparing the baseline score (D0) of this group, we found a higher frequency of score one and two at 6 and 12 months after treatments. The most likely straightforward explanation for these findings is that early after treatment the antigens released during the parasite killing maintain the protective effect on asthma severity. The effect of the antihelminthic treatments on the clinical score of asthma was perceived, however, only six to 12 months after repeated treatments. Considering that patients remained living in the same area using the contaminated water in their activities, we believe that only after sequential treatments, the loss of the protective effect of parasite infection over asthma symptoms could be observed. Although the asthma clinical score scale used in this study is not validated, against other measures of asthma severity or control, it gives an idea of the clinical course of the disease, when obtained repeatedly. It considers the use of antiasthmatic drugs and corticosteroids, as well as the need for the patient to visit emergency rooms or the hospital due to asthma attacks. The relevance of this score categorization was supported by the results of pulmonary function test which showed a significantly higher frequency of patients with abnormal lung function 12 and 18 months after antihelminthic treatments. For medical and ethical reasons, the group of placebo could not be left without antihelminthic treatment for more than 90 days. Hence, the two groups of patients received Albendazole and Praziquantel thereafter. One may ask if the adverse effect of long-term treatment on asthma severity we observed in this study would be due some seasonal differences during the follow-up study. We are aware that it is a limitation in this type of study design; however, as the study was conducted in a region with a tropical climate with no defined seasons and low weather variations during the year, this factor may not affect significantly the asthma severity.

Although our initial hypothesis in this study was that *S. mansoni* infection protects against asthma, the observation herein does not allow us to rule out the effect of other helminth infections in this protection, as have been proposed by other authors in experimental models of OVA-induced asthma [11–13]. In a polyhelminth endemic area, it is difficult to establish which parasite is protecting the host against a harmfully Th2-mediated pathological process. Previous systematic reviews and meta-analysis studying the effect of geohelminth infections on the risk of asthma showed that these parasites in general do not protect against asthma, but hookworm was shown to reduce the risk of the disease [14, 15]. A clinical trial using *Trichuris suis* ova resulted in no significant changes in symptom scores of allergic rhinitis [16]. Furthermore, treatment of hookworm infection, which leads to a reduction in the worm burden, increased the risk of allergen skin sensitization but did not interfere with the symptoms of allergic diseases [17], symptom scores of allergic rhinitis [16] and asthma [18]. In a systematic review and meta-analysis of epidemiological studies that researched the association between intestinal parasite infection and the presence of atopy, the authors found a consistent protective effect on allergic sensitization in patients with *Ascaris lumbricoides*, *Trichuris trichiura*, hookworm, or *Schistosoma* sp, infection [18]. There is evidence that in *S. mansoni* infection, the large number of regulatory cells and high levels of modulators molecules in the host system lead to a downmodulation not only of the parasite immune response, but also the immune response to bystander antigens as revised by Dunne and Cooke [19].

A possible explanation for these conflicting observations may include the time of exposure to the worm (if acute or chronic) and also the helminthic species. For instance, it was demonstrated in a randomized, double-blinded and placebo controlled trial that Albendazole and Praziquantel treatment of infected women during pregnancy was associated with increased risk of eczema and wheeze in the offspring [20]. New data presented here reinforce the idea that deworming may be associated with asthma worsenings. 

Regarding the effect of antihelminthic treatments on cytokine production, we were able to measure IFN-*γ*, IL-5, and IL-10 in response to Der p1 and to SWAP in supernatants of PBMC cultures before treatment and 7 and 90 days after treatment. Although Th2 molecules such as IL-4, IL-5, and IL-13 are key cytokines involved in the inflammatory response in asthma, IFN-*γ* has also been associated with asthma severity [21, 22]. The baseline levels of IL-5 in response to Der p1 in the present study were higher in the Praziquantel group compared to the placebo group. However, there was no significant difference in the pre- versus posttreatment levels of this cytokine in the two studied groups. On the other hand, the production of IL-10 in response to SWAP decreased 90 days after antihelminthic treatment in the Praziquantel group. This result suggests that the regulatory mechanisms exerted by the parasites begin to diminish early after treatment.

In the past few years, it has been shown that chronic helminth infections or parasite products induce the production of T-regulatory cells and molecules such as IL-10. This response has been associated with a down-modulation of allergic inflammatory mediators, such as Th2-cytokines, eosinophils, and histamine in murine models of allergic asthma [7, 12, 23]. We have characterized the *in vitro* immune response of asthmatic patients to some *S. mansoni* antigens and found a high production of *S. mansoni* antigen-specific IL-10 not only in cells of *S. mansoni* infected individuals but also in cells of noninfected asthmatic individuals [24]. There was also a significantly higher production of IL-10 and lower production of Th2-cytokine IL-5 in response to Der p1 in PBMC cultures of asthmatic patients infected with *S. mansoni* compared to non-infected asthmatic patients [25]. Our findings of decreased levels of IL-10 production after antihelminthic treatment in the present study, therefore, are in agreement with our's [25] and other authors, [26] previous studies.

Other regulatory mechanisms may contribute to the suppression of allergic inflammation induced by helminths. For instance, Pacífico et al. showed that T CD4^+^CD25^+^ cells protect mice against allergen induced airway inflammation through an IL-10 independent mechanism [12]. This finding differs from other studies, which have demonstrated that IL-10 is a key cytokine that suppresses the inflammatory response in OVA-induced asthma in mice infected with helminths. In mice infected with *H. polygyrus*, for example, the reduction in the number of eosinophils and in the levels of IL-5 was associated with IL-10 production and migration of regulatory cells to the draining lymph nodes [27].

It has also been demonstrated that cytotoxic T-lymphocyte antigen 4 (CTLA-4), a molecule rapidly upregulated after T-cell activation that provides negative feedback signaling, and limits the immune response as reviewed by Deurloo et al. [28], is involved in the suppression of allergic response in asthma [29]. We previously demonstrated that the lower levels of Th2-cytokines in asthmatics infected with *S. mansoni* compared to non-infected asthmatics were associated with a higher frequency of CD4^+^ T-cells expressing CTLA-4 [30]. Based on these findings, it is likely that the mechanisms underlying the regulation of inflammatory responses in asthma by *S. mansoni* antigens involve IL-10 [31], T-regulatory cells [12], and other mechanisms such as the expression of CTLA-4 [29, 30].

In the present study, where the number of asthmatic individuals who filled out the inclusion criteria and who agreed to participate was small, there was a significant worsening in the clinical scores of asthma as well as in the pulmonary function after repeated antihelminthic treatments. These findings are consistent with the hypothesis of the protective role of helminths on atopic diseases. Changes in clinical scores of asthma and in the pulmonary function tests were observed later after sequential antihelminthic treatments. Based on these results, we argue that the antigens released by the parasite when it dies can maintain protection against atopic diseases. After the clearance of antigens and repeated antihelminthic treatments to maintain a low parasite load, a loss of protection of parasite infection in the clinical course of asthma is observed. The data suggests that IL-10 could be implied in this protection. However, a better understanding of immune events following antihelminthic treatment is still required and may have practical consequences in the development of future therapies of allergic diseases. 

## Figures and Tables

**Table 1:** Baseline characteristics of studied subjects.

	Group 1 placebo *n* = 20	Group 2 Praziquantel *n* = 25	*P* value
Gender *n*			
Male *n* (%)	10 (50)	8 (32)	>0.05
Female *n* (%)	10 (50)	17 (68)	
Age group *n* (%)			
Child/teenagers (6–20 years old)	12 (65)	18 (72)	>0.05
Adult (21–50 years old)	8 (35)	7 (28)	>0.05
Rhinitis *n* (%)	10 (50)	18 (72)	>0.05
Smoking *n* (%)			
Active	6 (30)	7 (28)	>0.05
Passive	18 (90)	9 (36)	<0.005
Positive SPT response *n* (%)			
*Dermatophagoides pteronyssinus *	2 (10)	5 (20)	>0.05
*D. * *farinae *	2 (10)	5 (20)	>0.05
*Blomia tropicalis *	4 (20)	7 (28)	>0.05
*Periplaneta americana *	1 (5)	3 (12)	>0.05
*Blatella germanica *	2 (10)	3 (12)	>0.05
Positive total	9 (45)	9 (36)	>0.05
Current helminth infections *n* (%)			
*Schistosoma mansoni *	16 (80)	19 (76)	>0.05
*Ascaris lumbricoides *	10 (50)	14 (56)	>0.05
Hookworm	7 (35)	10 (40)	>0.05
*Trichuris trichiura *	10 (50)	13 (52)	>0.05
Coinfection (*S. * *mansoni* /1 or + helminths)	14 (90)	19 (100)	>0.05

**Table 2:** Levels of cytokines in supernatants of PBMC cultures in the studied subjects.

Cytokine	Antigen	D0		D7		D90	
		G1/placebo	G2/PZQ	G1/placebo	G2/PZQ	G1/placebo	G2/PZQ
IFN-*γ*	Without stimulus	32.7 (31.2–276.0)	31.6 (31.2–91.0)	31.2 (31.2–46.7)	39.7 (31.2–583.6)	31.2 (31.2–130.7)	32.7 (31.2–2563.0)
	SWAP	149.5 (42.0–10337.0)	88.0 (31.2–2092.0)	56.0 (43.8–1415.0)	137.8 (37.4–15543.0)	70.0 (15.6–3329.0)	130.7 (31.2–2563.0)
	Der p1	338.5 (102.4–7475.0)	710.0 (149.0–2568.0)	116.7 (42.0–420.2)	333.9 (32.7–9669.0)	201.0 (31.2–4818.0)	149.4 (15.6–1275.0)

IL-5	Without stimulus	15.6 (15.6–112.0)	15.6 (15.6-15.6)	15.6 (15.6-15.6)	15.6 (15.6–40.7)	15.6 (15.6–25.0)	15.6 (15.6–635.0)
	SWAP	468.5 (15.6–5156.0)	310.2 (15.6–5904.0)	542.0 (15.6–4444.0)	1863.0 (15.6–4606.0)	1670.0 (21.5–4444.0)	2850 (136.0–4444.0)
	Der p1	150.0 (15.6–630.0)^a^	463.0 (207.0–4606.0)^a^	28.8 (15.6–407.4)	60.0 (15.6–4606.0)	28.7 (15.6–1720.0)	352.0 (15.6–3509.0)

IL-10	Without stimulus	27.5 (15.6–475.0)	28.0 (15.6–102.0)	57.0 (16.8–589.0)	23.1 (15.6–1144.0)	36.0 (15.6–446.9)	15.6 (15.6–127.0)
	SWAP	264.5 (26.0–1688.0)	642.0 (125.0–1132.0)^b^	288.0 (29.4–641.0)	667.3 (15.6–2447.0)	240.0 (21.1–638.4)	175.6 (60.3–1062.0)^b^
	Der p1	451.0 (56.0–1060.0)	349.0 (177.0–1539.0)	924.0 (297.1–2063.0)	288.0 (15.6–2086.0)	390.0 (54.3–1744.0)	204.0 (60.0–638.0)

Data showed as median (minimum and maximum values) pg/ml;

^a^Placebo D0 versus PZQ D0 *P* < 0.05 (Mann-Whitney *t*-test);

^b^PZQ D0 versus PZQ D90 *P* < 0.05 (Wilcoxon matched-pairs signs ranks test).
